# Design of a Multi-Ion Detection System Based on IoT Technology and Its Application in Cement-Based Materials

**DOI:** 10.3390/s26123933

**Published:** 2026-06-20

**Authors:** Yudong Sun, Zijing Zhang, Yixuan Li, Shaoyang Ding, Hanbo Chen, Zhengeng Xu, Yuejing Li, Xincheng Li, Dafu Wang, Jun Ren

**Affiliations:** 1 School of Architecture and Planning, Yunnan University, Kunming 650091, China; sunyudong@stu.ynu.edu.cn (Y.S.); zhangzijing1@stu.ynu.edu.cn (Z.Z.); liyixuan@stu.ynu.edu.cn (Y.L.); imdingdingking@163.com (S.D.); 17387062046@163.com (H.C.); zhengengxu@163.com (Z.X.); m15803402215@163.com (Y.L.); wangdafu92@163.com (D.W.); renjunking@aliyun.com (J.R.); 2 Yunnan Institute of Building Research, Kunming 650223, China; 3 Yunnan Key Laboratory of Building Structure and New Materials, Kunming 650223, China; 4 Yunnan Key Laboratory of Carbon Neutrality and Green Low-Carbon Technologies, Yunnan University, Kunming 650050, China

**Keywords:** multi-ion in situ detection, ion-selective electrodes, cement-based materials, IoT sensing, leaching kinetics

## Abstract

**Highlights:**

**What are the main findings?**

- Successfully developed a highly integrated, low-cost IoT system for synchronous multi-parameter (Cl^−^, Ca^2+^, F^−^, and pH) in situ detection, achieving a minimum resolvable concentration of 10^−5^ M with good short-term repeatability.
- Applied the system to phosphogypsum–cement matrices, successfully tracking the multi-ion competitive synergy process involving rapid Ca^2+^ leaching, simultaneous F^−^ precipitation consumption, and non-monotonic Cl^−^ release on a unified timeline.

**What are the implications of the main findings?**

- Overcomes the limitations of traditional single-point discrete sampling by directly obtaining inter-ionic correlation information through multi-parameter collaborative testing.
- Provides a practical sensing platform for assessing degradation mechanisms, evaluating material durability, and providing early warning for environmental risks in cement-based solid waste utilization.

**Abstract:**

Simultaneous multi-ion detection is important for interpreting leaching, corrosion, hydration, and solidification processes in cement-based materials, because these processes are controlled by coupled ion migration, binding, and precipitation–dissolution reactions. Conventional methods such as pore-solution extraction, ion chromatography, inductively coupled plasma optical emission spectroscopy, and single-ion potentiometric measurements provide useful chemical information, but they generally rely on discrete sampling or isolated ion channels and therefore have limited ability to capture time-aligned multi-ion evolution. In this study, an IoT-based in situ multi-ion detection system was developed by integrating ion-selective electrodes for Cl^−^, Ca^2+^, F^−^, and H^+^ with an ADS1115 analog-to-digital converter, an ESP32 microcontroller, and a voltage amplification module. The system achieved minimum resolvable concentrations of 10^−5^ M for Cl^−^ and F^−^ and 10^−4^ M for Ca^2+^, while maintaining pH measurement over the range of 2–12. Ten consecutive measurements at 0.01 M showed relative standard deviations below 0.12%, indicating good short-term repeatability under laboratory calibration conditions. Interference and temperature tests showed that Br^−^ and NO_3_^−^ affected the chloride channel at high concentrations, Ca^2+^ reduced free F^−^ activity through Ca–F precipitation equilibrium, and the temperature drift of Cl^−^ and F^−^ electrodes changed direction with concentration, whereas the Ca^2+^ response decreased monotonically with increasing temperature. When applied to phosphogypsum–cement hardened pastes, the system captured rapid Ca^2+^ release, low-level F^−^ fluctuation controlled by Ca–F interaction, non-monotonic Cl^−^ release, and alkaline pH evolution on the same time axis. Compared with existing single-ion or offline methods, the proposed system provides synchronized in situ evidence for interpreting coupled ion leaching in cement-based solid-waste systems.

## 1. Introduction

Cement-based materials are among the most widely used engineering materials in modern infrastructure, and their production, service performance, and maintenance are closely related to carbon emissions, resource consumption, and structural safety [1,2,3]. In recent years, low-carbon binders and the large-scale utilization of industrial solid wastes have been regarded as important approaches for reducing clinker consumption, extending service life, and decreasing environmental burdens [2,3,4,5]. However, the transition toward low-carbon and solid-waste-based cementitious materials is not only a matter of simple mixture replacement. With the incorporation of mineral admixtures, industrial by-product gypsum, solidified waste matrices, and complex service media, the ionic composition, equilibrium relationships, and transport processes in cement-based pore solutions may continuously change. These changes can further affect the stability of hydration products, the risk of steel corrosion, leaching safety, and long-term durability [6,7,8]. Therefore, the engineering application of low-carbon cement-based materials requires not only sufficient strength and workability, but also continuous monitoring and mechanistic understanding of the chemical evolution of their liquid phases.

The durability degradation of cement-based systems is essentially the result of multi-ion migration and reaction. Chloride ingress can destroy the passive film on reinforcing steel and induce localized corrosion, but the initiation of corrosion is not determined by Cl^−^ concentration alone; it is also closely related to OH^−^ activity, pH, temperature, and the surface condition of steel [9,10,11,12]. Sulfate ions can react with aluminate and calcium phases in cement-based materials, changing the chloride-binding capacity and promoting the formation of expansive products [13,14]. Continuous calcium leaching can lead to the dissolution of portlandite and the decalcification of C–S–H, thereby increasing porosity, weakening the continuity of the matrix, and altering subsequent ion diffusion pathways [15,16,17]. For phosphogypsum–cement systems, the problem becomes more complicated. Soluble fluoride, phosphate, sulfate, and trace impurities in phosphogypsum may undergo dissolution, adsorption, precipitation, and re-dissolution in highly alkaline pore solutions. In particular, the fixation of F^−^ through the formation of CaF_2_ or aluminum-bearing hydration phases can directly influence the environmental risk and long-term stability of the solidified matrix [18,19,20,21]. These processes do not occur independently. Instead, they share key reactive components such as Ca^2+^, OH^−^, SO_4_^2−^, Cl^−^, and F^−^ in the same pore solution. Therefore, single-ion and discrete concentration data are insufficient to reveal the competition, delay, and feedback relationships among different ions.

Current methods for characterizing the liquid-phase chemistry of cement-based materials mainly include pore solution expression, leaching extraction, ion chromatography, ICP-OES/ICP-MS, titration, and spectroscopic analysis [6,22,23,24]. These offline methods have high accuracy in standardized testing and endpoint concentration analysis, and they have provided substantial basic data for understanding hydration reactions and pore solution composition [6,22]. However, their limitations are also evident. Samples usually need to be removed, filtered, diluted, acidified, or otherwise pretreated, and the obtained results essentially represent quantitative information at a specific sampling time. For early hydration, rapid ion exchange, sudden changes during the initial leaching stage, and long-term leaching thresholds, discrete sampling often cannot provide sufficient temporal resolution [23,24]. Recently, dynamic filtration combined with high-time-resolution ICP-OES has been used to capture the early evolution of cement pore solutions. Nevertheless, such methods are still mainly suitable for short-term laboratory analysis and are difficult to directly transform into long-term, low-cost, in situ, and multi-point monitoring systems for engineering applications [24].

Electrochemical sensing technology offers a possible way to overcome this limitation. Ion-selective electrodes (ISEs) have the advantages of simple structure, rapid response, relatively low cost, and suitability for in situ measurement. They have been used for detecting Cl^−^, pH, and corrosion-related parameters in cement-based materials [25,26,27,28,29,30,31]. Ag/AgCl chloride electrodes, solid-state chloride sensors, thick-film pH sensors, and methods for simultaneous Cl^−^/pH monitoring of corrosion thresholds have demonstrated the potential of potentiometric sensors in concrete durability assessment [25,26,27,28,29,30]. Meanwhile, multi-parameter corrosion sensors have attempted to integrate chloride concentration, pH, electrical resistivity, corrosion current, and temperature into a single monitoring unit [31,32]. However, most existing studies still focus on Cl^−^ or pH monitoring in steel corrosion scenarios, and the number of target ions remains limited. In addition, under high ionic strength, temperature fluctuation, and multi-ion interference conditions, reference electrode drift, liquid junction potential variation, membrane aging, and cross-response can significantly affect measurement reliability [26,33,34].

At the same time, the development of solid-contact ISEs, ISE arrays, multivariate signal decoupling algorithms, and IoT-based embedded platforms is promoting the transition from single-probe measurement to integrated, networked, and intelligent ion detection [35,36,37,38,39]. Previous studies have shown that ISE arrays combined with multivariate calibration, artificial neural networks, or proportional factor models can simultaneously resolve multiple ions in natural water, agricultural irrigation solutions, and environmental samples, while partially overcoming non-ideal selectivity and cross-interference [37,38,39]. Low-cost microcontrollers such as ESP32 have also been widely used for IoT nodes, wireless communication, and real-time data transmission, providing a hardware basis for field-oriented, multi-node, and long-term monitoring [40,41]. However, cement pore solutions usually have high ionic strength, complex coexisting anions and cations, and a continuously changing buffer system during hydration and leaching. To date, there is still a lack of a system-level sensing platform specifically designed for cement-based liquid environments that can synchronously collect key parameters such as Cl^−^, Ca^2+^, F^−^, and pH on the same time axis, while also evaluating its applicability under temperature disturbance, ion interference, and actual leaching conditions of solid-waste-based cementitious materials.

Based on the above research gap and our previous work on automated sulfate titration and IoT-based testing systems for cement-based materials [42,43,44], this study designs and constructs a simultaneous multi-ion in situ detection system for cement-based liquid environments. The novelty of this study lies in three main aspects. First, different from previous studies mainly focusing on single Cl^−^ or pH sensing, this work integrates Cl^−^, Ca^2+^, and F^−^ combination ion-selective electrodes with a pH electrode into a four-parameter sensing array, and realizes synchronous acquisition of weak potential signals through an ESP32 microcontroller, dual ADS1115 analog-to-digital converters, and voltage amplification modules. Second, this study systematically investigates the different effects of temperature fluctuation and typical coexisting ions on ISE responses, revealing that the temperature-drift direction of Cl^−^ and F^−^ electrodes can reverse with concentration variation, while the Ca^2+^ electrode exhibits a negative temperature-drift response. Third, the proposed system is applied to dynamic leaching experiments of phosphogypsum–cement composite solidified matrices, where it synchronously captures rapid Ca^2+^ release, F^−^ precipitation consumption, non-monotonic Cl^−^ release, and pH evolution on a unified time axis. This verifies its potential value for environmental risk assessment, durability mechanism analysis, and early warning in the utilization of solid-waste-based cementitious materials.

## 2. Test Principles and Instrument Design

### 2.1. Test Principles

Using multiple combination ion-selective electrodes and a pH electrode, the system enables simultaneous measurement of various ions (Cl^−^, Ca^2+^, F^−^) and pH in a solution. The sensor output signals are filtered and amplified by a voltage amplification module, then uniformly acquired by a multi-channel analog-to-digital converter (ADC). Data is processed in the main control unit at a preset sampling frequency, printed to the serial port, or uploaded in real time to a cloud server via a WiFi communication module in packaged data packets. The computer connected to the main control board automatically interprets the received voltage sequences into real-time concentration values for each ion. When the system requires calibration, the voltage values can also be printed directly.

In this measurement workflow, the millivolt-level potential signals generated by different ion-selective electrodes are incorporated into a unified acquisition and conversion process. Through multi-channel voltage acquisition, channel-specific calibration, temperature-dependent concentration conversion, and unified time-series recording, the system enables synchronized in situ detection of Cl^−^, Ca^2+^, F^−^, and pH. Compared with single-ion offline analysis or independent sensor readouts, this system can obtain multi-parameter variation trends within the same experimental process, providing a data basis for analyzing ion migration, precipitation, and mutual interactions during the leaching of cement-based materials.

### 2.2. Hardware System

#### 2.2.1. Combination Ion-Selective Electrodes and Their Operating Principles

Composite electrodes are a class of electrochemical functional materials that achieve synergistic electron and ion conduction by incorporating ionic conductors or solid-state electrolyte components into a conductive matrix. Their operating principle is based on the electrochemical reaction kinetics and ion migration mechanisms at the electrode-electrolyte interface, enabling the conversion of energy or matter through the transport of electrons and ions within a multiphase system. Under an applied potential, electrons migrate along the metal or carbon framework within the conductive phase, while ions migrate within the embedded or coated ionic conductor driven by concentration gradients and electric field forces. This process is typically described by the Nernst-Planck equation:

(1) $$J_{i}=-D_{i}\nabla c_{i}-\frac{z_{i}u_{i}F}{RT}c_{i}\nabla \phi$$

In the equation, $J_{i}$ is the flux of ion species i (mol·m^−2^·s^−1^), $D_{i}$ is the diffusion coefficient (m^2^·s^−1^), $\nabla c_{i}$ is the concentration gradient (mol·m^−4^), $z_{i}$ is the valence of the ion, $u_{i}$ is the mobility (m^2^·V^−1^·s^−1^), F is the Faraday constant (C·mol^−1^), R is the gas constant (J·mol^−1^·K^−1^), T is the temperature (K), and ∇ϕ is the potential gradient (V·m^−1^).

Under steady-state transmission conditions, the motion of electrons can be described by Ohm’s law:

(2) $$J_{e}=\sigma E$$

In the equation, $J_{e}$ is the current density in the electron-conducting phase (A·m^−2^), σ is the electrical conductivity of the electron-conducting phase (S·m^−1^), and E is the electric field strength (V·m^−1^).

Electrochemical reactions occur at the interface between electron and ion flows within the electrode; the reaction rate is governed by the interfacial overpotential and can typically be described by the Butler-Volmer equation:

(3) $$j=j_{0}\left[\exp\left(\frac{\alpha_{a}F\eta }{RT}\right)-\exp\left(-\frac{\alpha_{c}F\eta }{RT}\right)\right]$$

In the equation, j is the current density, $j_{0}$ is the exchange current density, $\alpha_{a}$ and $\alpha_{c}$ are the charge transfer coefficients in the anode and cathode directions, respectively, and η is the overpotential (V).

The key feature of a combination ion-selective electrode is the integration of an indicator electrode (ISE), which selectively responds to specific ions, with a built-in reference electrode that provides a stable potential reference, all within a single housing. This creates a self-contained measurement system that enables potentiometric detection of ion activity without the need for an external reference electrode. Structurally, a combination ion-selective electrode typically consists of an indicator electrode unit, a reference electrode unit, and a housing. At the core of the electrode is the sensing membrane, which possesses the ability to specifically recognize and respond to the target ion. When the sensing membrane comes into contact with the solution under test, a membrane potential $E_{m}$ is formed across the membrane due to the selective adsorption, diffusion, or ion exchange of ions. The magnitude of the membrane potential follows the Nernst equation and can be expressed as:

(4) $$E_{m}=E^{0}+\frac{2.303RT}{nF}log\frac{a_{i,\mathrm{ext}}}{a_{i,\mathrm{int}}}$$

In the equation, $E^{0}$ is the standard potential of the reference electrode (which depends on the electrode material, the characteristics of the sensing membrane, and temperature); R is the ideal gas constant (8.314 J·mol^−1^·K^−1^); T is the thermodynamic temperature (K); n is the charge of the target ion; F is the Faraday constant (96,485 C·mol^−1^); $a_{i,\mathrm{ext}}$ is the activity of the target ion in the solution under test; $a_{i,\mathrm{int}}$ is the activity of the target ion in the internal solution of the reference electrode. Since $a_{i,\mathrm{int}}$ and $E^{0}$ remain constant during the detection process, the membrane potential $E_{m}$ depends solely on the activity $a_{i,\mathrm{ext}}$ of the target ion in the sample solution, i.e., $E_{m}\propto a_{i,\mathrm{ext}}$. This is the fundamental principle underlying quantitative ion detection.

When performing the measurement, the formula can be simplified to:

(5) $$E=E^{0}+\frac{2.303RT}{nF}\log_{10}\left(c_{i}\right)$$

In the equation, $c_{i}$ represents the concentration of ion i (mol/L).

The Nernst equation provides a quantitative relationship between potential and ionic activity, but the selectivity of an electrode depends entirely on the material properties of the sensing membrane. Different target ions require specific membrane-phase recognition mechanisms.

**Figure 1:**
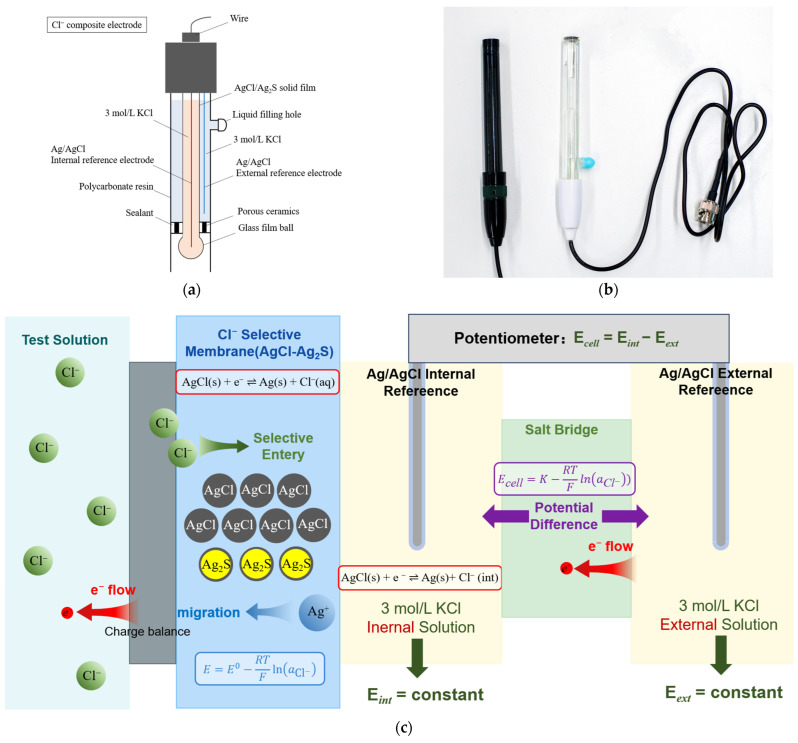
Chloride ion electrode: (**a**) Internal structure; (**b**) Actual specimen; (**c**) Mechanism. Note: In AgCl-Ag_2_S membrane, Ag^+^ is the mobile ion (not Cl^−^). Solid arrows = primary flux; Purple = measured potential. All potentials referenced to standard hydrogen electrode (SHE).

The core of the chloride ion combination electrode is an AgCl–Ag_2_S mixed-phase sensing membrane. This material utilizes Ag_2_S doping to create Ag^+^ lattice migration channels, while Cl^−^ participates solely in interfacial equilibrium, endowing the membrane with the ability to specifically recognize Cl^−^. When the sensing membrane comes into contact with the test solution, an interfacial ion exchange equilibrium occurs on the membrane surface: AgCl(s) ⇌ Ag^+^(mem) + Cl^−^(aq). Changes in the Cl^−^ activity in the test solution directly modulate the interfacial Ag^+^ chemical potential, driving the directed migration of Ag^+^ within the membrane. A stable interfacial potential and charge separation layer are established on both sides of the membrane/solution interface due to the difference in Cl^−^ activity, thereby enabling potentiometric detection as illustrated in Figure 1.

**Figure 2:**
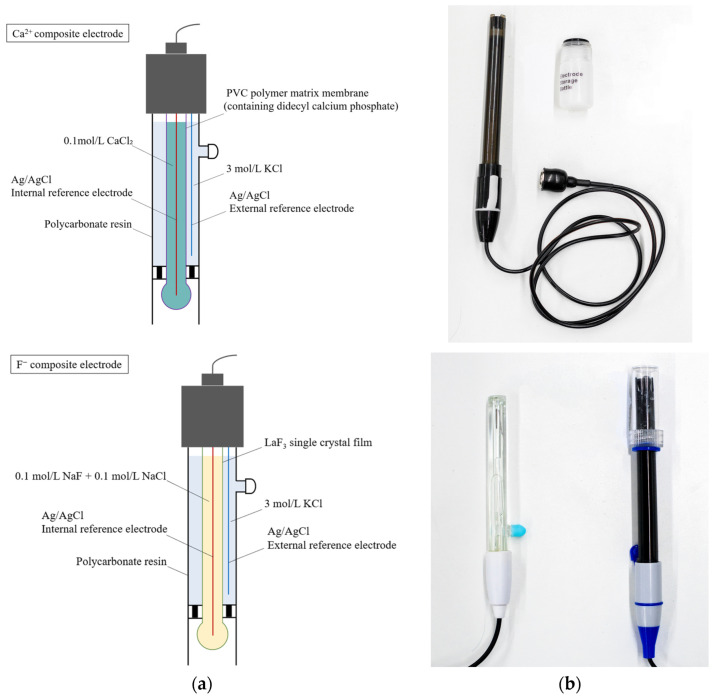
Calcium ion combination electrode and fluoride ion combination electrode: (**a**) internal structure; (**b**) actual specimen.

The core of a calcium ion combination electrode is a polymeric sensing membrane that acts as a Ca^2+^-selective carrier, typically composed of a PVC matrix doped with calcium di-decyl phosphate and plasticizers. This carrier forms stable and reversible complexes with Ca^2+^, endowing the membrane with the ability to specifically recognize Ca^2+^. It exhibits high coordination selectivity and strongly inhibits the conduction of coexisting cations such as Na^+^, K^+^, and Mg^2+^ due to differences in binding constants. When the membrane comes into contact with the test solution, Ca^2+^ in the solution migrates toward the membrane surface driven by a concentration gradient and undergoes selective ion exchange through complexation and dissociation with the carrier, with its structure and operational principle depicted in Figure 2.

The core of the fluoride ion composite electrode is a single-crystal sensitive film of europium-doped (Eu^2+^) lanthanum fluoride (LaF_3_). Pure LaF_3_ has low ionic conductivity; however, when divalent cations are doped to replace La^3+^ in the lattice, a large number of F^−^ vacancies are created to maintain electrical neutrality. These vacancies form dedicated channels for the rapid hopping conduction of F^−^ within the lattice. This crystal structure exhibits high lattice compatibility with F^−^, while coexisting anions such as Cl^−^, SO_4_^2−^, and NO_3_^−^ face significant conduction barriers due to mismatched radii and charges, thereby ensuring excellent selectivity. Upon activation, a phase boundary distribution equilibrium for F^−^ is rapidly established at the membrane/liquid interface, and a stable phase boundary potential difference forms on both sides of the membrane due to differences in F^−^ activity.

**Figure 3:**
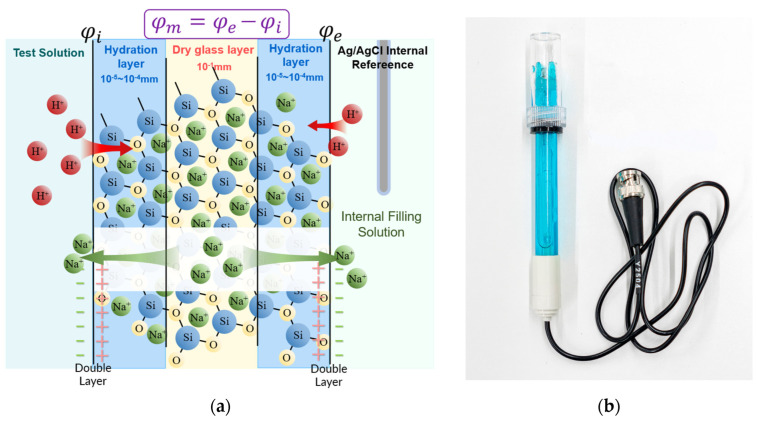
pH electrode (**a**) Mechanism; (**b**) Actual specimen.

The core of a pH composite electrode is a specialized silicate glass sensing membrane. As shown in Figure 3, after the glass membrane is activated, an extremely thin hydrated gel layer forms on its surface, with a dry glass layer beneath it. During operation, Na^+^ in the hydrated layer on the outer side of the membrane undergoes reversible ion exchange with H^+^ in the sample solution; since the Si–O– network in the silicate structure has a much stronger binding affinity for H^+^ than for Na^+^, the exchange equilibrium is strictly controlled by the H^+^ activity in the outer solution. Similarly, an identical ion exchange equilibrium is established between the hydrated layer on the inner side of the membrane and the internal filling solution. Due to the difference in H^+^ activity, interface potentials $\varphi_{i}$ and $\varphi_{e}$ are formed at the two interfaces, respectively. The intermediate dry glass layer serves only to conduct Na^+^ charge and does not alter the thermodynamic equilibrium at the interfaces. The total transmembrane potential $\varphi_{m}=\varphi_{e}$ − $\varphi_{i}$ is determined solely by the ratio of H^+^ activities between the sample solution and the internal filling solution, exhibiting a linear response characteristic that is inversely proportional to the pH value:

(6) $$E=E^{0}-\frac{2.303RT}{F}\cdot \mathrm{pH}$$

The following electrodes were selected for the experiment: the E-301 pH composite electrode (Shanghai INESA Scientific Instrument Co., Ltd., Shanghai, China), the PCL-201-C chloride ion composite electrode (Shanghai Shuaiying Industrial Co., Ltd., Shanghai, China), the PF-1C fluoride ion composite electrode (Shanghai Shuaiying Industrial Co., Ltd., Shanghai, China), and the 7101 calcium ion electrode (Shanghai Russell Technology Co., Ltd., Shanghai, China).

#### 2.2.2. Voltage Amplifier Module

**Figure 4:**
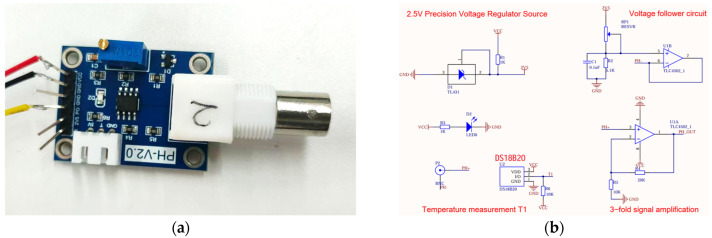
Voltage amplifier module: (**a**) 3D model; (**b**) circuit diagram.

The raw potential signals generated by the sensing membrane are typically weak DC voltages in the millivolt range and are susceptible to interference from temperature drift and non-ideal interface factors. To achieve high-precision data acquisition, the signals must undergo signal conditioning and system-level calibration. The system’s voltage operational amplifier uses the Gravity EC Meter transmitter module manufactured by Luoyang Guantuo Electronics Technology Co., Ltd., Luoyang, China, with a gain of G as shown in Figure 4. The voltage amplification module is used to condition the millivolt-level weak potential signals generated by the ion-selective electrodes, making them more suitable for subsequent ADS1115 analog-to-digital conversion and digital recording. Considering the differences among electrodes in response slope, zero potential, and low-concentration signal fluctuation, the system does not directly use the raw potential values for concentration determination. Instead, the measured potentials are further converted according to channel-specific calibration curves and the corresponding temperature conditions. This treatment improves the consistency of data interpretation among different ion channels.

In practical detection systems, due to variations in electrode manufacturing processes, aging of the sensing membrane, drifts in the Nernst slope and intercept caused by fluctuations in ambient temperature, and the zero-point bias voltage of the signal conditioning circuit, a comprehensive system correction factor α is introduced to apply an engineering correction to the theoretical model in order to improve accuracy. The corrected system output voltage model is:

(7) $$E=\left(GE^{0}-\alpha \right)+G\frac{2.303RT}{nF}\log_{10}c_{i}$$

(8) $$E=\left(GE^{0}-\alpha \right)-G\frac{2.303RT}{F}\cdot \mathrm{pH}$$

#### 2.2.3. Main Control Board

The main control board serves as the core control and data processing unit of the multi-ion detection system. It is built on the ESP32-DevKit V1 microcontroller module (Espressif Systems Co., Ltd., Shanghai, China), which integrates the ESP32-WROOM-32 chip. This microcontroller features a dual-core 240 MHz Tensilica LX6 processor architecture capable of handling real-time multitasking and parallel computing, and supports both Wi-Fi and Bluetooth communication. The circuit employs a single-sided PCB layout. The left side primarily houses the ESP32 module and power supply circuitry, while the right side accommodates dual ADS1115 (Texas Instruments, Dallas, TX, USA) data acquisition modules and peripheral interfaces for connecting multiple voltage amplifier modules. The ESP32 architecture has demonstrated excellent deployment reliability when building low-cost environmental monitoring nodes [22], effectively ensuring network connectivity and data packet integrity during long-term communication [23].

To support multi-channel synchronous detection, a dual-channel ADS1115 high-precision analog-to-digital converter (ADC) architecture is employed. These converters communicate with the main control chip via an I^2^C bus to enable high-precision analog signal acquisition across eight independent channels. Each ADS1115 features 16-bit resolution and a maximum sampling rate of 860 SPS, capable of acquiring weak electrical signals with a theoretical sampling accuracy of ±0.256 mV. The main control program initializes two independent I^2^C channels, corresponding to different pin groups (SDA and SCL), ensuring the synchronization and accuracy of multi-channel signals and providing reliable raw data for ion concentration conversion based on the Nernst equation.

For power supply, the board uses a dedicated DC step-down module to regulate the external 12 V input into two voltage rails—5 V and 3.3 V—which power the ESP32, the ADS1115 modules, and pins UA0 through UA7. The onboard power selection jumper and power switch allow the power supply to be adjusted based on the power consumption or logic level requirements of the peripherals.

Upon power-up, the ESP32 first performs connection detection and operating mode configuration for the two ADS1115 chips, including setting the sampling rate, input mode, and programmable gain amplifier parameters. During the sampling phase, the dual ADCs simultaneously acquire analog signals and convert them into voltage data.

**Figure 5:**
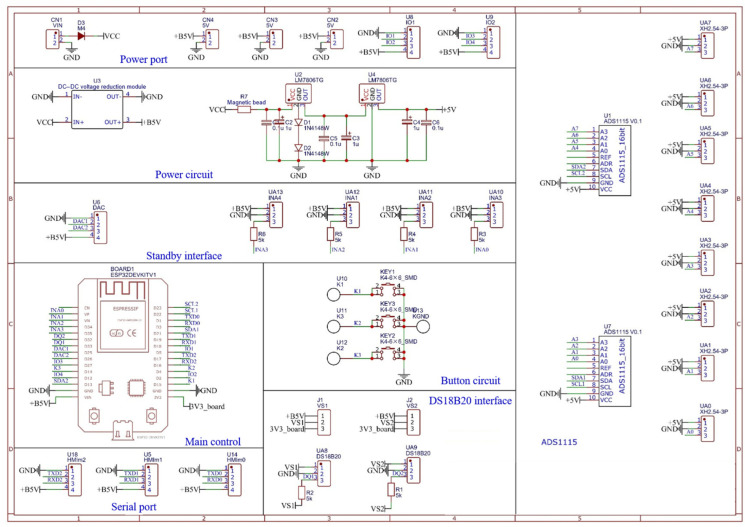
Component circuit.

**Figure 6:**
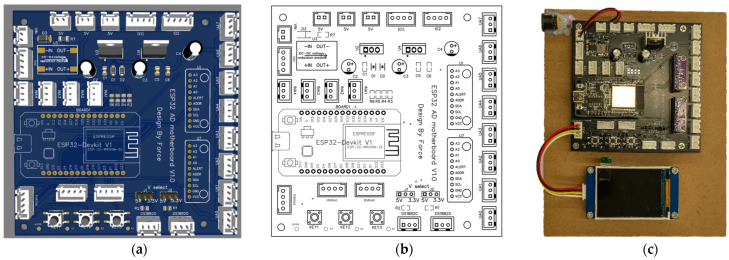
Main control board (**a**) 3D Model; (**b**) Silkscreen layer; (**c**) Physical prototype.

In addition, the main control board features GPIO ports, dual I^2^C interfaces, a UART serial port, a DS18B20 temperature sensor interface, and interfaces for buttons and a display. Three user-programmable buttons (KEY1, KEY2, and KEY3) can be used for data acquisition and control, parameter debugging, or function switching. The circuit layout and the physical implementation of the main control board are presented in Figure 5 and Figure 6, respectively.

#### 2.2.4. Power Supply and System Assembly

**Figure 7:**
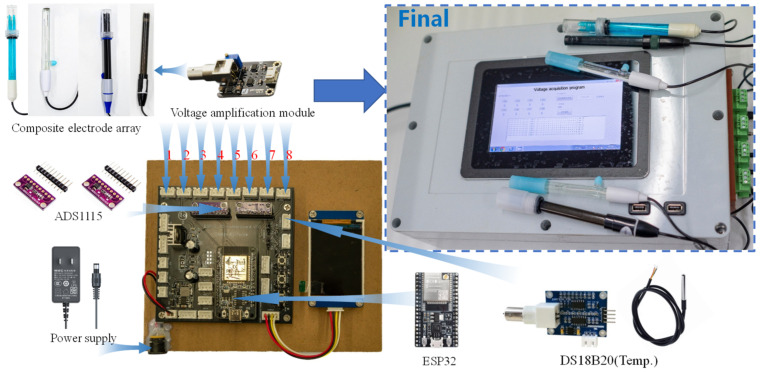
Assembly diagram.

The power supply unit selected for the experiment is a regulated DC power adapter, model: PPI-E57-12V02A. The input is compatible with standard AC 220 V, 50 Hz mains power and can be directly connected to a conventional power circuit; the output provides a stable DC voltage of 12 V with a rated output current of 1000 mA, which is connected to the main control board. The final system assembly is shown in Figure 7.

### 2.3. Software System

The program was written using the Arduino IDE (version 1.8.13, Arduino SA, Turin, Italy) platform, implemented in C/C++, and uploaded to the ESP32-DevKit V1 development board (Espressif Systems (Shanghai) Co., Ltd., Shanghai, China). During system startup, the program automatically configures the I2 C communication interface, initializes the dual ADS1115 analog-to-digital conversion modules, identifies their addresses, and sets the sampling frequency and input channels. During the sampling phase, the ESP32 reads the voltage signals from each electrode channel at the preset sampling frequency. The data obtained within the same acquisition cycle are organized as one multi-parameter data frame, including the voltage responses of Cl^−^, Ca^2+^, F^−^, and pH channels.

Although the channel signals are acquired through the ADC and I2C bus in a sequential reading process, the data are assigned to a unified acquisition sequence during storage and analysis. This data-frame-based organization enables time alignment among different ion and pH channels, allowing the variation trends of Cl^−^, Ca^2+^, F^−^, and pH to be compared on the same time axis. This software-level synchronization is important for analyzing the coupled evolution of multiple ions during the leaching of cement-based materials.

The system includes a built-in Wi-Fi module that connects to the OneNet cloud platform. It uses the MQTT protocol to upload encapsulated data packets in real time. The platform assigns channels to the uploaded data and adds timestamps, providing functions such as curve display and historical data storage. This study focuses primarily on the detection component, while wireless monitoring capabilities can be further expanded in future developments.

## 3. Calibration of the Measurement System

### 3.1. Data Selection, Accuracy, and Stability

**Figure 8:**
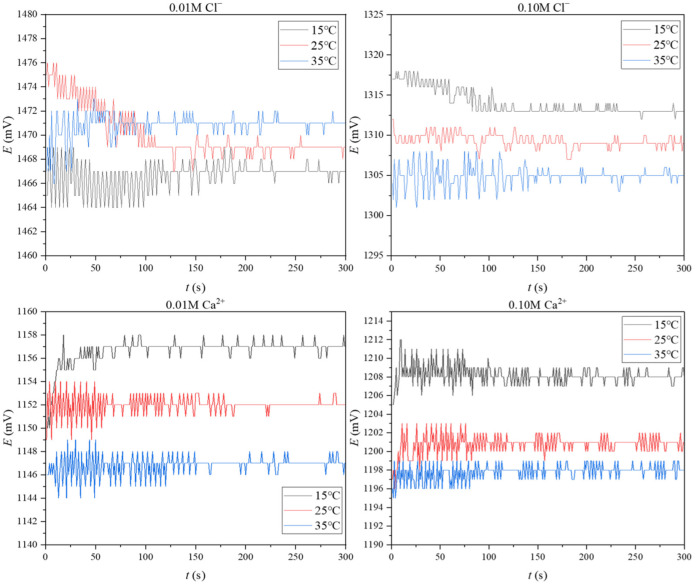
Potentiometric response curve.

Calibration experiments typically involve three temperature gradients: 15 °C, 25 °C, and 35 °C. Temperature control is achieved using a constant-temperature water bath with a control accuracy of better than ±0.5 °C. The water level in the bath is maintained at the same level as the liquid surface in the container holding the sample. Additionally, a DS18B20 temperature probe is inserted into the leaching solution to record the actual liquid temperature. Figure 8 shows the system’s potentiometric response curve. The system exhibits a significant response to changes in the concentration of the corresponding ions. During the initial measurement phase (t < 50 s), the potential signal displays notable high-frequency oscillations or drift, reflecting the non-equilibrium state resulting from the rapid reorganization of the double layer at the electrode–solution interface and the initial stage of ion diffusion. Over time, the system gradually transitions toward thermodynamic equilibrium, and the response signal exhibits clear signs of stabilization. Signals at different temperatures all retain varying degrees of dynamic fluctuation. After t > 120 s, the mean potentials under all test conditions tend to stabilize, providing continuous and repeatable measurement signals that meet the detection requirements.

**Figure 9:**
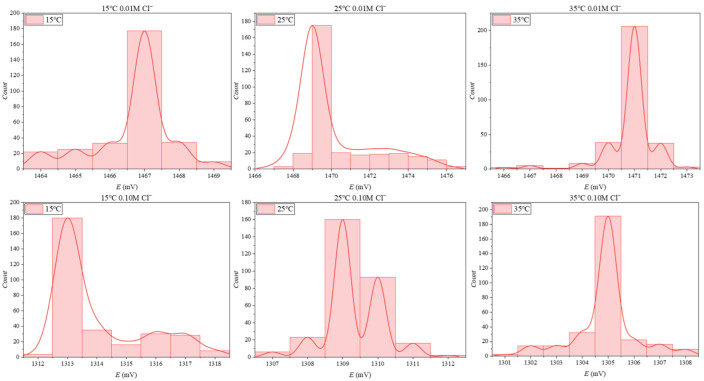
Data distribution.

Figure 9 shows the histogram of the statistical distribution of the potentiometric responses at different temperatures for a fixed concentration, with a sampling frequency of 1 Hz and a total duration of 300 s. The potential data exhibit a distinct unimodal distribution over the measurement period. Gaussian fitting revealed that, following a transient response, the sensors oscillate primarily around a specific central potential value. In this study, the peak of the distribution curve was selected as the final measured potential.

**Figure 10:**
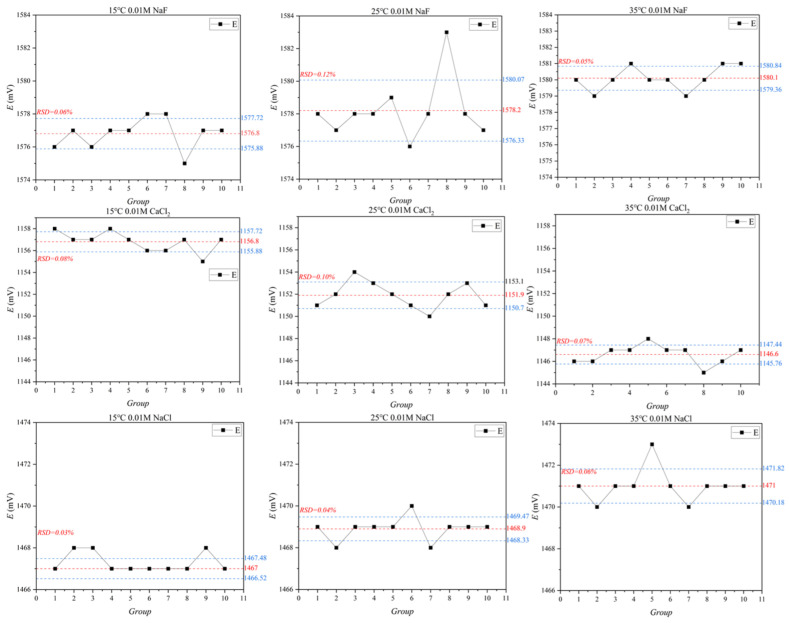
Stability testing at 25 °C with 0.01 M 10-fold series. Red dashed lines indicate the mean potential values; blue dashed lines indicate the upper and lower bounds.

As shown in Figure 10, stability testing revealed that, based on 10 consecutive test runs, the system’s RSD for 0.01 M solutions was consistently below 0.12%, primarily ranging between 0.03% and 0.08%, indicating a low coefficient of variation.

Although the ADS1115 analog-to-digital converter used in this system has a theoretical resolution of 16 bits and a least significant bit (LSB) of approximately 7.8125 μV, the actual measurement accuracy is affected by the ion-selective electrode, the amplification circuit, reference electrode stability, and interfacial mass-transfer processes at low ion concentrations. Therefore, experimental calibration is required to evaluate the practical resolving capability of the system. As the target ion concentration enters the low-concentration range, the ion-exchange flux at the membrane-solution interface decreases markedly, and the electrode response gradually deviates from ideal Nernstian behavior. Meanwhile, the steady-state potential exhibits stronger random fluctuations in the time domain. Consequently, the measurement error bar increases as the concentration decreases. In the medium-to-high concentration range, the error bars remain small, and the system exhibits a high signal-to-noise ratio and good concentration resolution. In the low-concentration range, although the error bars increase significantly, adjacent concentration changes can still be distinguished as long as the potential uncertainty remains smaller than the potential difference corresponding to the adjacent concentration gradient.

In this study, the error bar was obtained by extracting the raw potential data over 300 s and calculating the standard deviation $\sigma_{E}$, and the minimum resolvability criterion was based on $3\sigma_{E}$. It should be noted that this criterion represents a resolvable threshold in the potential domain and should not be regarded as a constant error limit in the concentration domain. For the calibration relationship $E=A+S\;\log_{10}c$ the concentration can be back-calculated as $c=10^{\left(\left(E-A\right)/S\right)}$. Therefore, the relative uncertainty propagated from the potential standard deviation to the concentration domain can be approximated as $\sigma_{c}/c=\ln\left(10\right)\sigma_{E}/\left|S\right|$. When the 3σ criterion is used, the expanded relative uncertainty is approximately $3\ln\left(10\right)\sigma_{E}/\left|S\right|$. This indicates that, in the low-concentration range, the back-calculated concentration error is further amplified when the response slope decreases, the steady-state potential fluctuation increases, or the response intervals of adjacent concentration levels begin to overlap. Based on this consideration, the critical point at which $3\sigma_{E}$ equals or exceeds the potential difference corresponding to the adjacent concentration gradient is defined here as the actual lower limit of resolvable concentration under the present hardware and calibration conditions, rather than a strict quantitative limit of detection.

**Figure 11:**
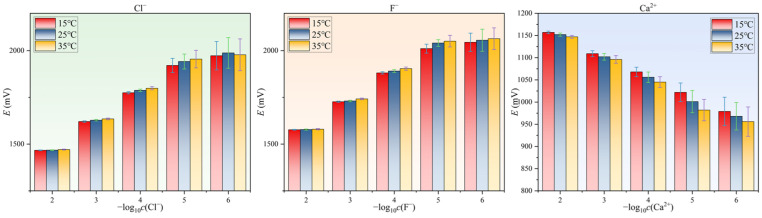
Determination of the actual lower limit of resolvable concentration for Cl^−^, F^−^, and Ca^2+^ based on the 3σ potential uncertainty criterion.

As summarized in Figure 11, the actual lower limits of resolvable concentration under the present experimental conditions are 10^−5^ M for Cl^−^, 10^−5^ M for F^−^, and 10^−4^ M for Ca^2+^, respectively.

### 3.2. Testing of Standard Titrant Solutions

#### 3.2.1. pH Calibration

At 298 K (25 °C), tests were conducted using standard pH 6.86, 4.00, and 9.18 buffers, and NaOH and HCl solutions ranging from 10^−5^ M to 10^−2^ M were prepared for calibration. The electrodes were also calibrated at 15 °C and 35 °C by varying the temperature.

In the ideal Nernst equation, the potential of the pH electrode and the pH value form a single straight line across the entire measurement range; however, in practical applications, the response of the pH electrode is non-ideal due to acid and base offsets in combination glass electrodes during actual measurements, causing the electrode response to deviate from the ideal Nernst slope; the asymmetric potential of the electrode and the liquid junction potential of the reference electrode exhibit inconsistent behavior in the strong acid and strong base regions, failing to follow a single straight line. The module incorporates a signal amplification circuit, which amplifies the range deviation of the electrode’s raw signal in a synchronized manner, ultimately resulting in the separation of the fitting lines into acidic, neutral, and alkaline regions.

**Figure 12:**
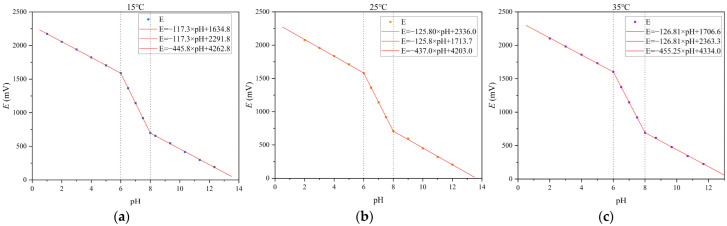
pH calibration curves: (**a**) 15 °C; (**b**) 25 °C; (**c**) 35 °C.

The following fit was obtained, as shown in Figure 12, revealing a good linear relationship within the pH detection range of 2 to 12:

#### 3.2.2. Chloride Ion Calibration

**Figure 13:**
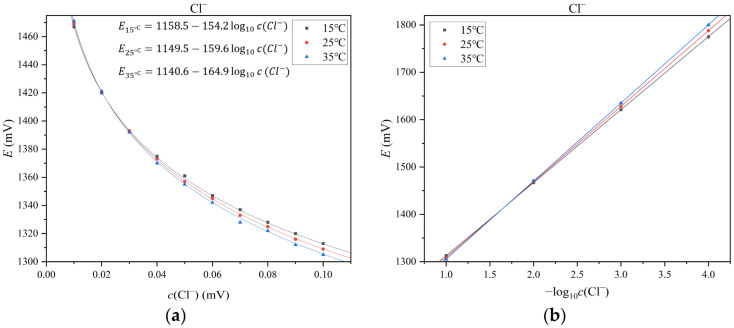
Fitting curves for the chloride ion electrode: (**a**) Fitting relationship for 0.01–0.10 M; (**b**) Logarithmic fitting relationships for 0.1, 0.01, 0.001, and 0.0001.

Figure 13 shows the static calibration curves of the chloride ion detection unit at 15 °C, 25 °C, and 35 °C. The plots of concentration versus potential E versus $-{log}_{10}c({\mathrm{Cl}}^{-})$ are shown, and the fitting equations are as follows:

$$E_{15\;{}^{\circ}C}=1158.5-154.2\log_{10}c({Cl}^{-});R^{2}=0.9997$$

$$E_{25\;{}^{\circ}C}=1149.5-159.6\log_{10}c({Cl}^{-});R^{2}=0.9999$$

$$E_{35\;{}^{\circ}C}=1140.6-164.9\log_{10}c({Cl}^{-});R^{2}=0.9995$$

Combining Equation (7), the absolute values of the slope of the fitted line—154.2, 159.6, and 164.9 mV/decade—correspond to the system’s equivalent gain term $G\frac{2.303RT}{nF}$. Within the temperature range of 15 °C to 35 °C, the system’s actual hardware gain G remains constant at approximately 2.70. The slope increases linearly with rising temperature, following the Nernst response law. Furthermore, the fitted intercept decreases linearly with rising temperature, corresponding to a temperature drift coefficient of approximately −0.90 mV/°C. This baseline shift may originate from the inherent negative temperature characteristics of the built-in reference system and the thermal evolution of the liquid junction potential.

#### 3.2.3. Fluoride Ion Calibration

**Figure 14:**
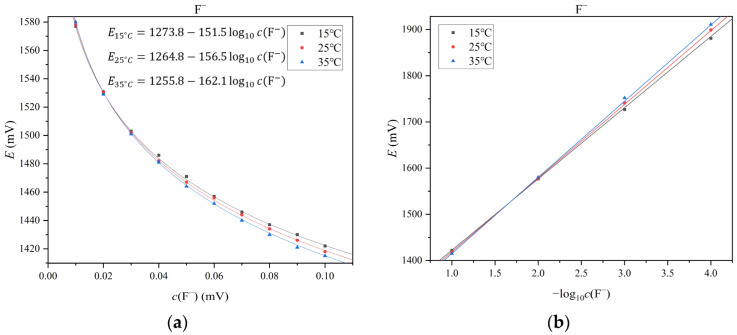
Fitting curves for fluoride ion electrodes: (**a**) Fitting relationship for 0.01–0.10 M; (**b**) Logarithmic fitting relationships for 0.1, 0.01, 0.001, and 0.0001.

Figure 14 shows the calibration curves for the fluoride ion detection unit at three temperatures. The fitted equations are as follows:

$$E_{15\;{}^{\circ}C}=1273.8-151.5\log_{10}c\left(F^{-}\right);R^{2}=0.9996$$

$$E_{25\;{}^{\circ}C}=1264.8-156.5\log_{10}c\left(F^{-}\right);R^{2}=0.9997$$

$$E_{35\;{}^{\circ}C}=1255.8-162.1\log_{10}c\left(F^{-}\right);R^{2}=0.9998$$

The fitted intercept of the fluoride ion combination electrode is significantly higher than that of the chloride ion combination electrode by approximately 115 mV; this may be due to the strong affinity of the LaF_3_ lattice for F^−^ and the high ionic conductivity of the single-crystal membrane, which together result in a unique potential reference for the fluoride ion combination electrode.

#### 3.2.4. Calcium Ion Calibration

**Figure 15:**
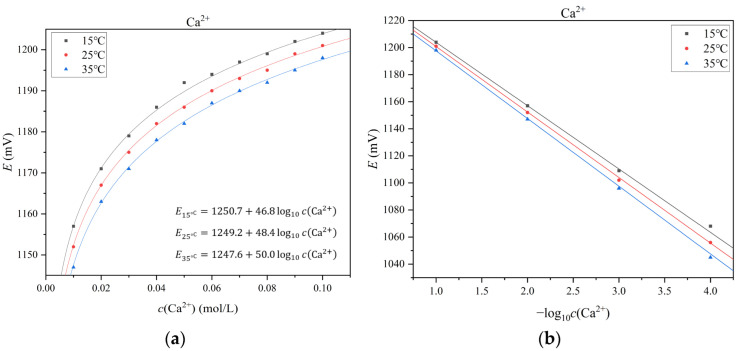
Fitting curves for calcium ion electrodes: (**a**) Fitting relationship for 0.01–0.10 M; (**b**) Logarithmic fitting relationships for 0.1, 0.01, 0.001, and 0.0001.

Figure 15 shows the static calibration curves of the calcium ion detection unit at three temperatures. The fitted equations are as follows:

$$E_{15\;{}^{\circ}C}=1250.7+46.8\log_{10}c\left({\mathrm{Ca}}^{2+}\right);R^{2}=0.9975$$

$$E_{25\;{}^{\circ}C}=1249.2+48.4\log_{10}c\left({\mathrm{Ca}}^{2+}\right);R^{2}=0.9990$$

$$E_{35\;{}^{\circ}C}=1247.6+50.0\log_{10}c\left({\mathrm{Ca}}^{2+}\right);R^{2}=0.9991$$

G remains relatively stable in the range of 15–35 °C; the fitted intercept decreases linearly with increasing temperature, and the temperature drift coefficient is approximately −0.31 mV/°C. In the PVC-based polymer sensing membrane of the calcium ion combination electrode, the complexation reaction between Ca^2+^ and the calcium di-decyl phosphate carrier is an exothermic process; an increase in temperature reduces the complexation stability constant and weakens the ion exchange capacity at the membrane interface. Compared to the chloride and fluoride ion combination electrodes, the absolute value of the calcium ion intercept is higher, possibly due to the intrinsic differences between the PVC carrier of the sensing membrane and the reference system.

The above calibration results are comparable with, and in some aspects complementary to, previously reported potentiometric methods for cementitious environments. Ag/AgCl chloride electrodes have been shown to exhibit Nernstian response in alkaline solutions and mortar, but their practical detection limit can be affected by the configuration of the reference electrode [10,45]. In the present system, the actual lower limits of resolvable concentration reached 10^−5^ M for Cl^−^ and F^−^ and 10^−4^ M for Ca^2+^ under the current hardware, amplification, and calibration conditions. These values are suitable for the leaching experiment in Section 5, where F^−^ remained at the 10^−5^ M level and Cl^−^ was mainly in the 10^−4^–10^−3^ M range. Compared with studies focused on a single chloride channel, the present system emphasizes the use of channel-specific calibration curves to obtain time-aligned Cl^−^, Ca^2+^, F^−^, and pH information within the same measurement sequence [27,28,30].

The short-term repeatability test also helps clarify the applicable boundary of the system. Ten consecutive measurements at 0.01 M produced RSD values below 0.12%, indicating low random fluctuation under controlled laboratory conditions. However, this result should be interpreted as short-window repeatability rather than long-term embedded durability. Existing reviews and long-term studies of Ag/AgCl sensors show that membrane aging, liquid-junction potential and depletion or absence of chloride near the sensing surface can affect sensor stability in concrete environments [10,30,46]. Therefore, the calibration results support the use of the proposed system for laboratory in situ leaching analysis, while long-term field deployment still requires additional drift and durability evaluation.

## 4. Factors Affecting Measurement Performance

### 4.1. Interfering Ions Test

#### 4.1.1. Effect of Interfering Ions on Chloride Detection

**Figure 16:**
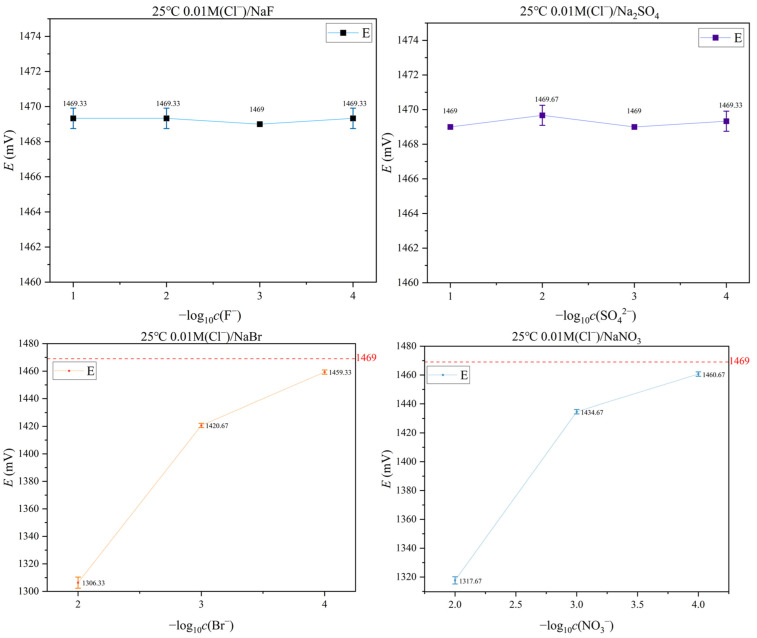
Effect of interfering ions on chloride ion testing. Error bars represent the standard deviation of repeated steady-state measurements.

Under conditions of 25 °C and a fixed background Cl^−^ concentration of 0.01 mol/L, the effects of four typical anions—SO_4_^2−^, F^−^, Br^−^, and NO_3_^−^—on the chloride combination electrode potentiometric response at different concentration gradients were investigated. The results in Figure 16 show that within a wide concentration range where −log_10_c(SO_4_^2−^) and −log_10_c(F^−^) ranged from 1 to 4, the electrode potential remained stable at approximately 1469 mV, which is highly consistent with the reference potential of a pure 0.01 mol/L Cl^−^ solution. Across the entire tested concentration range, SO_4_^2−^ and F^−^ did not effectively compete for ion exchange sites on the surface of the electrode’s sensing membrane, and their interfering effects were negligible.

Br^−^ and NO_3_^−^ exhibit significant concentration-dependent interference with the chloride combination electrode; as the concentration of interfering ions increases, the electrode potential shifts markedly in the negative direction. When the concentrations of Br^−^ and NO_3_^−^ reach 0.01 mol/L, the potential shift caused by NO_3_^−^ and Br^−^ is substantial; when the concentrations of both ions drop to 10^−4^ mol/L, the electrode potentials recover to 1459 mV and 1460 mV, respectively, with the deviation from the reference potential being less than 10 mV in both cases, making the interference effect essentially negligible. This drastic change in potential is due to Br^−^ competing with Cl^−^ for membrane sites because its solubility product is close to that of AgCl; at high concentrations, NO_3_^−^ alters the ionic strength of the system, thereby affecting the structure of the double layer at the membrane interface and leading to the generation of a mixed potential.

#### 4.1.2. Effect of Interfering Ions on Calcium Detection

**Figure 17:**
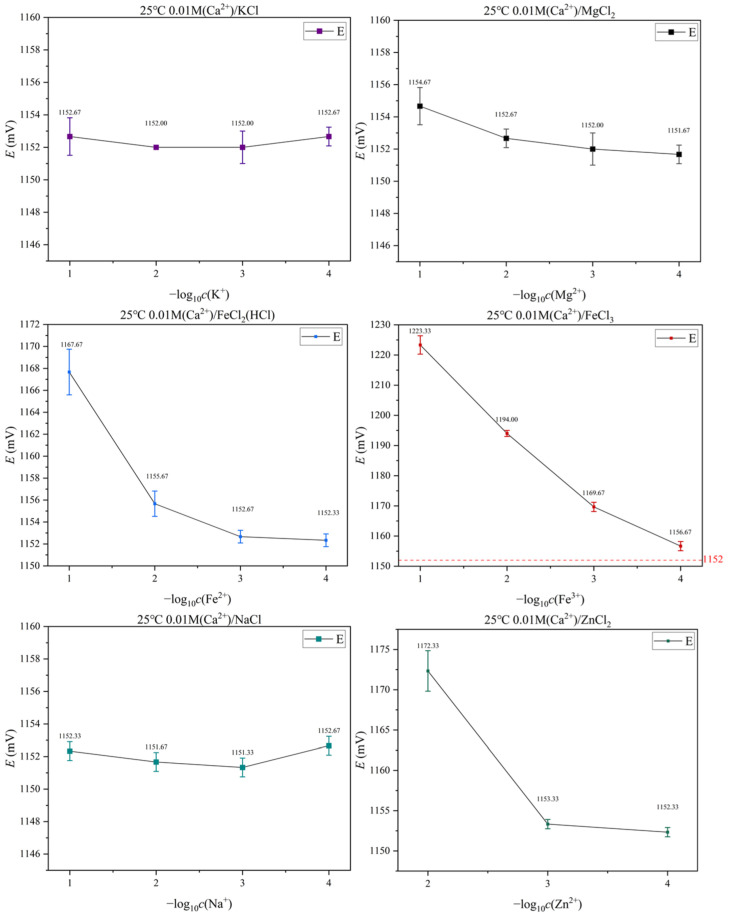
Effect of interfering ions on calcium ion testing. Error bars represent the standard deviation of repeated steady-state measurements.

Under conditions of 25 °C and a fixed background Ca^2+^ concentration of 0.01 mol/L, the effects of Na^+^, K^+^, Mg^2+^, Zn^2+^, Fe^2+^, and Fe^3+^ on the potential response of the calcium combination electrode were investigated, as presented in Figure 17.

In tests involving the typical coexisting cations Na^+^, K^+^, and Mg^2+^ in cement-based materials, the electrode potential remained stable within the range of 1151.33–1152.67 mV over a wide concentration range of −log_10_c(M^n+^) from 1 to 4, with negligible interference. As the concentrations of Zn^2+^, Fe^2+^, and Fe^3+^ ions increase, the electrode potential exhibits a significant positive shift; however, when the concentration drops below 10^−4^ mol/L, the potential gradually returns to near the baseline value, and the interference effect can be largely ignored.

#### 4.1.3. Effect of Interfering Ions on Fluoride Determination

**Figure 18:**
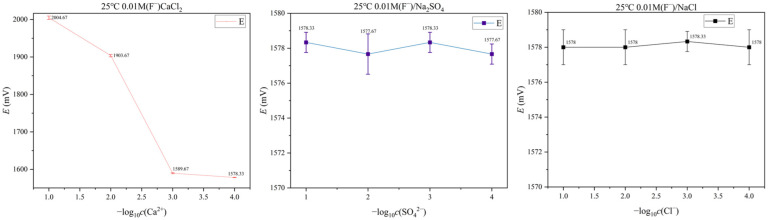
Effect of interfering ions on fluoride ion testing. Error bars represent the standard deviation of repeated steady-state measurements.

Similarly, in the fluoride ion interference test (Figure 18), the potential of the fluoride ion combination electrode remained stable near the 1578 mV baseline for both Cl^−^ and SO_4_^2−^, indicating that the interference was negligible. However, when the Ca^2+^ concentration increases, a significant positive shift occurs, and the potential value drifts substantially. This phenomenon is not due to selective competitive interference of Ca^2+^ with the LaF_3_ single-crystal membrane, but rather results from Ca^2+^ complexing with free F^−^ to form insoluble CaF_2_ (Ksp ≈ 3.9 × 10^−11^), thereby reducing the activity of free F^−^ in the system under test. In cement-based hardened bodies, the curing/leaching behavior of F^−^ is regulated by the Ca–F precipitation equilibrium; the system’s sensitivity to changes in F^−^ activity can be used to track the phase transformation and leaching processes of fluorides in situ.

The interference behavior observed in this study is consistent with the selectivity issues reported for potentiometric sensors in cementitious environments. For chloride detection, previous studies have emphasized that Ag/AgCl ISEs can be affected by hydroxide activity and the chemical environment of cement pore solutions [10,45,46]. In the present test, SO_4_^2−^ and F− produced negligible interference with the chloride channel, whereas Br^−^ and NO_3_^−^ caused concentration-dependent potential shifts. This indicates that the chloride channel is suitable for the phosphogypsum–cement leaching system investigated here, where Br^−^ and NO_3_^−^ are not dominant species, but it also suggests that additional correction or recalibration would be necessary if the same system were applied to solutions rich in competing monovalent anions.

For Ca^2+^ and F^−^, the interference results also agree with the chemistry of cementitious and phosphogypsum-bearing systems. Calcium ISEs typically show selectivity against common cations such as Na^+^, K^+^, and Mg^2+^, although the final response depends on membrane composition, ionophore behavior, and conditioning history [47]. In the present study, Na^+^, K^+^, and Mg^2+^ produced negligible disturbance to the Ca^2+^ channel, whereas Zn^2+^, Fe^2+^, and Fe^3+^ caused stronger shifts at high concentrations. For F^−^ detection, the most important effect was not direct anion competition but the decrease in free F^−^ activity caused by Ca^2+^. This is consistent with the expected Ca–F precipitation or stabilization mechanism in phosphogypsum-containing cementitious systems [20,21,48].

### 4.2. Effect of Temperature

#### 4.2.1. Effect of Temperature on Chloride Detection

**Figure 19:**
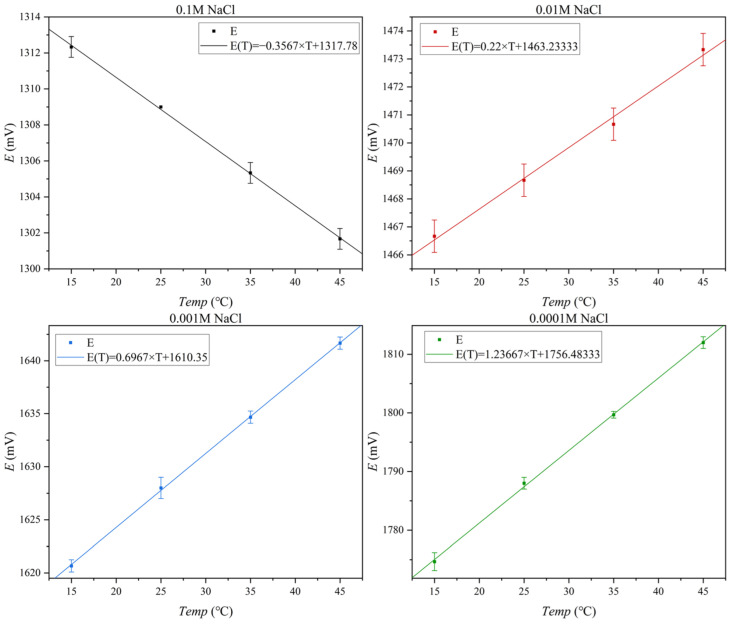
Chloride ion detection is affected by temperature.

Figure 19 shows that the potentiometric response of the chloride ion combination electrode exhibits significant temperature- and concentration-dependence. Within the test range of 15–45 °C, the electrode potential in a 0.1 mol/L NaCl solution drifts linearly in the negative direction as the temperature increases, with a temperature coefficient of approximately −0.36 mV/°C. However, in the low-concentration range of 0.01–0.0001 mol/L, the potentiometric response shifts to a positive drift, and the temperature coefficient increases from 0.22 mV/°C to 1.24 mV/°C as the concentration decreases, exhibiting a clear reversal in the sign of the temperature drift. Based on the extended Nernst equation $E=E{}^{\circ}\left(T\right)+\frac{RT}{F}\ln a_{{\mathrm{Cl}}^{-}}$, an increase in temperature theoretically increases the slope of the Nernst response, driving a positive shift in the potential; under high-concentration conditions, the activity coefficient of chloride ions decreases significantly with rising temperature; combined with the inherent negative temperature coefficients of the Ag/AgCl internal reference potential and the porous liquid junction potential, this ultimately dominates the macroscopic negative temperature drift. In the dilute solution range, the change in ionic activity with temperature slows down, and the temperature effect on the Nernst slope becomes the core controlling factor of the potentiometric response. At the same time, rising temperature reduces the ion exchange activation energy at the membrane–solution interface and accelerates interfacial mass transfer kinetics, amplifying the positive potentiometric response at low concentrations; this effect becomes increasingly pronounced as the concentration is diluted. For this section, steady-state repeated error bars have been selected.

#### 4.2.2. Effect of Temperature on Fluoride Detection

**Figure 20:**
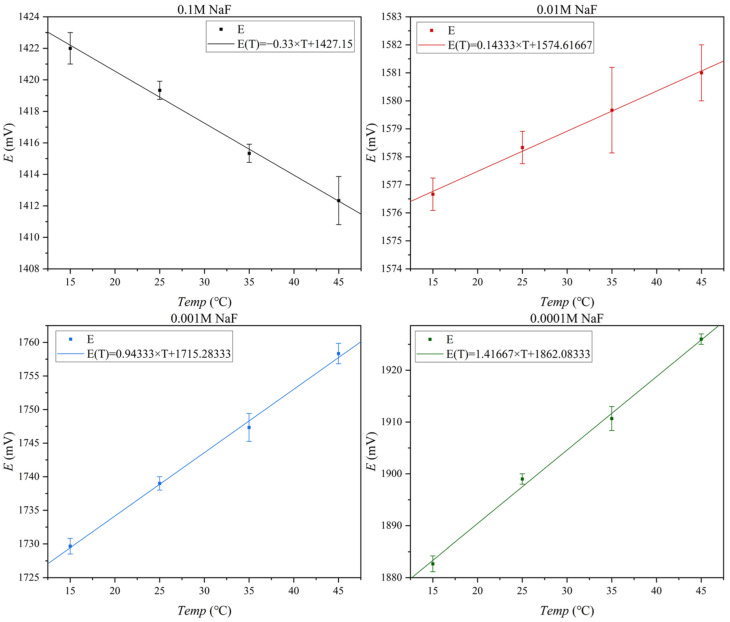
Fluoride ion detection is affected by temperature.

The temperature response of the fluoride ion combination electrode is concentration-dependent, as indicated in Figure 20, exhibiting a trend similar to that of the chloride ion combination electrode but with a different mechanism. Within the test range of 15–45 °C, the electrode potential in a 0.1 mol/L NaF solution exhibits a linear negative drift with increasing temperature, with a temperature coefficient of −0.43 mV/°C; when the F^−^ concentration is diluted to the range of 0.01–0.0001 mol/L, the direction of the temperature drift reverses, and the temperature coefficient surges from 0.14 mV/°C to 1.423 mV/°C, exhibiting pronounced positive temperature sensitivity. At high concentrations, the negative temperature effect of the reference potential and the liquid junction potential dominates the drift; at low concentrations, the positive temperature drift is driven jointly by the thermal activation characteristics of the LaF_3_ membrane and the Nernst effect.

#### 4.2.3. Effect of Temperature on Calcium Detection

**Figure 21:**
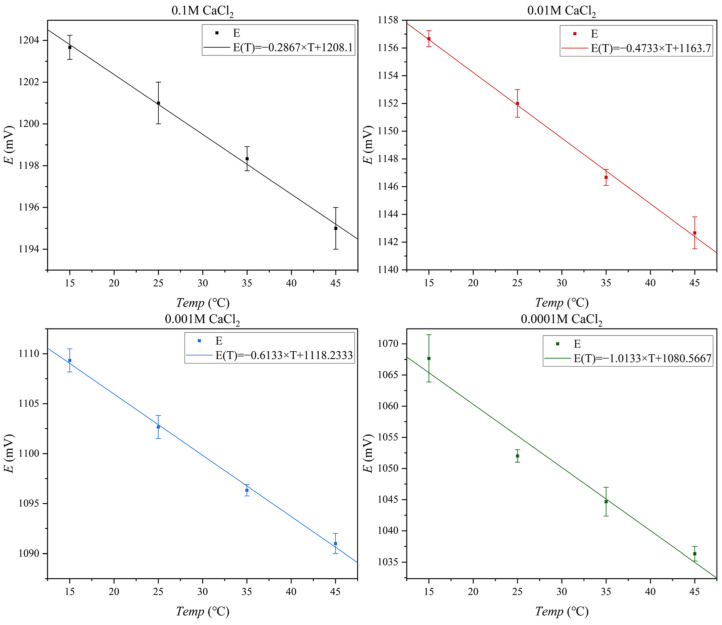
Calcium ion detection is affected by temperature.

The temperature response of the calcium ion combination electrode exhibits significant concentration dependence. Within the range of 15–45 °C and 0.1 mol/L to 0.0001 mol/L, as presented in Figure 21, the electrode potential decreases monotonically and linearly with increasing temperature, without any reversal of the temperature drift sign; the absolute value of the temperature coefficient increases stepwise from 0.29 mV/°C to 1.01 mV/°C. This phenomenon stems from the combined effects of multiple factors: in a divalent cation system, the slope of the Nernst response increases with rising temperature; at low concentrations, the logarithm of the activity term is negative, driving the potential term toward a more negative value; simultaneously, in the PVC-based neutral carrier membrane used for the calcium ion combination electrode, the complexation reaction between Ca^2+^ and the carrier is an exothermic process, which significantly reduces the complexation stability constant, promotes complex dissociation, and weakens the effective ion-exchange capacity at the membrane interface, further lowering the phase boundary potential. At high concentrations, this effect combines with the intrinsic negative temperature coefficient of the internal reference system and that of the liquid junction potential to form a fundamental negative temperature drift. As the concentration is diluted, the influence of the Nernst thermodynamic term and carrier dissociation is continuously amplified, causing the absolute value of the temperature coefficient to increase progressively as the concentration decreases.

The temperature-response results further show that a single temperature coefficient is insufficient for multi-ion potentiometric detection in cement-based leachates. For Ag/AgCl chloride sensors, temperature affects both the Nernst slope and the reference/sensing interface, while cement pore solutions introduce additional complexity through ionic strength, pH, and liquid-junction effects [10,30,46]. In the present system, the Cl^−^ and F^−^ electrodes exhibited a concentration-dependent reversal in the direction of temperature drift: high-concentration solutions showed negative drift, whereas dilute solutions showed positive drift. By contrast, the Ca^2+^ channel showed a monotonic decrease in potential with increasing temperature, which is consistent with the temperature sensitivity of carrier-based Ca^2+^ membranes and the thermodynamic influence of Ca^2+^ complexation/dissociation [47,49].

## 5. Application in Cement-Based Materials

### 5.1. Experimental Design

To verify the system’s applicability and measurement stability in the detection of environmental responses in actual cement-based materials, this section describes a dynamic leaching experiment on a phosphogypsum-cement-based hardened paste. The experiment employs a controlled-variables approach to systematically investigate the effects of different phosphogypsum content and ambient temperature on the leaching kinetics of characteristic ions (F^−^, Cl^−^, Ca^2+^) and pH evolution.

#### 5.1.1. Sample Preparation and Experimental Groups

The experimental materials consisted of ordinary Portland cement (P·I 52.5) produced by Qingdao Shanshui Innovation Cement Co., Ltd. (Qingdao, China) and industrial phosphogypsum. The phosphogypsum used was a washed phosphogypsum supplied by Yunnan Xiangfeng Industrial Group Co., Ltd. (Anning, China), with a median particle size (d_50_) of 27.4 μm, predominantly composed of CaSO_4_·2H_2_O and exhibiting a plate-like crystal morphology. Prior to the experiment, the phosphogypsum was dried in an oven at (40 ± 2) °C to constant weight to remove residual moisture and then sieved through a 0.3 mm square-mesh sieve; the cement was used directly without additional treatment. The water-to-binder ratio of all specimens was uniformly controlled at 0.4. After being cast into standard molds, the fresh pastes were cured under constant temperature and humidity conditions (temperature 20 ± 2 °C, relative humidity ≥ 95%) following the standard cement curing regime for 28 days. After curing, the hardened pastes were crushed and ground, and then sieved through a 1.18 mm square-mesh sieve to eliminate the influence of particle size differences on the leaching and mass transfer processes. Five experimental groups (G1–G5) were established based on the phosphogypsum content (mass percentage), and the specific mix proportions are shown in Table 1.

#### 5.1.2. Leaching System Setup and Environmental Control

The leaching experiments were conducted in a sealed, temperature-controlled reaction vessel. For each group, 10 g of crushed test specimens with a particle size of <1.18 mm was weighed. Deionized water was added at a solid-to-liquid ratio of 1:10 (g/mL), and the mixture was allowed to stand for leaching to establish a homogeneous leaching system. The experiment was conducted at three temperature gradients: 15 °C, 25 °C, and 35 °C. Temperature control was achieved using a constant-temperature water bath with a control accuracy of better than ±0.5 °C. The water level in the bath was maintained at the same level as the liquid surface in the vessel to ensure uniform heat exchange. During measurements, a DS18B20 temperature probe was inserted into the leaching solution to record the actual liquid temperature.

#### 5.1.3. Measurement Parameters and Data Acquisition Strategy

The system performs in situ measurements with the electrode probe suspended 2 cm below the liquid surface to avoid contact with precipitates. The system records the response potentials of Cl^−^, Ca^2+^, F^−^, and pH in time-aligned acquisition frames at a frequency of 1 Hz. To account for both the initial rapid dissolution phase and the subsequent slow equilibrium phase, a non-equidistant sampling strategy was employed, with time points at 15, 30, 45, and 60 min; 3 and 6 h; and days 1 through 7. Under each temperature condition, concentration conversions were performed using the corresponding temperature-specific calibration curves, and the mean ion concentrations and pH values were calculated for each time point.

### 5.2. Ion Leaching Analysis and System Validation

#### 5.2.1. pH Evolution

**Figure 22:**
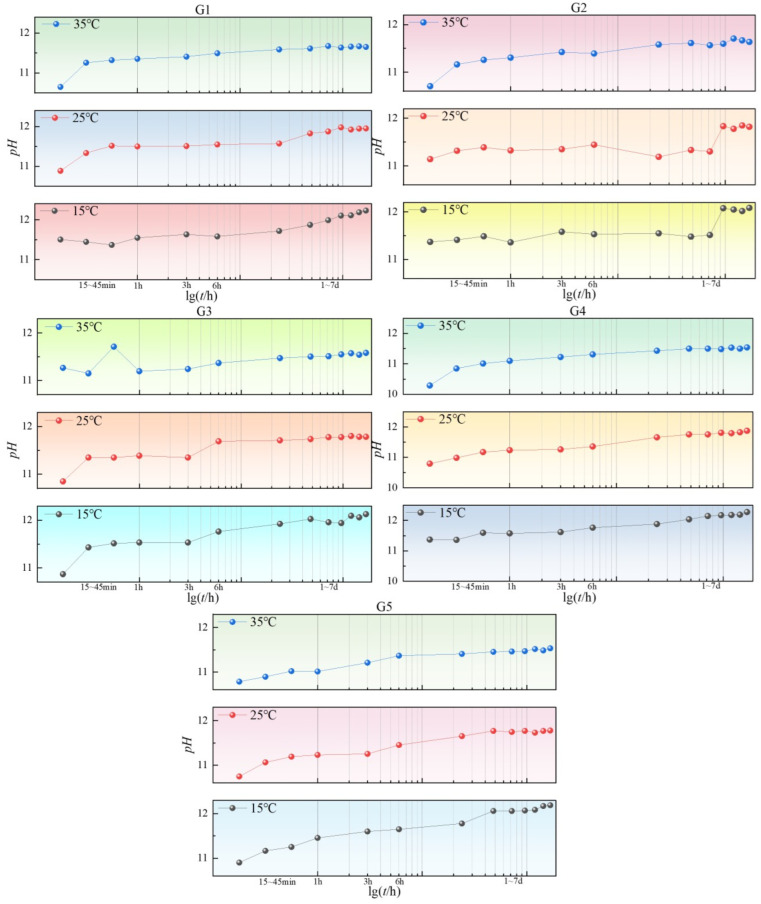
pH changes in the leaching solutions over time.

Figure 22 shows that the pH values in all experimental groups initially rose rapidly and then gradually stabilized. During the initial leaching period (15–45 min), the pH values ranged from 10.1 to 11.3 across all groups. Notably, the high-blending groups G4 and G5 exhibited lower initial values under certain temperature conditions, which is attributed to the early consumption of hydroxide ions by residual acidic impurities in the phosphogypsum; as leaching time increased, the differences among the groups gradually decreased, and after 7 days, all groups stabilized within the range of 11.8–12.3.

The system can perform measurements in environments where the pH exceeds 11.5 without showing any significant fluctuations or baseline drift, indicating good stability under highly alkaline conditions. During the initial leaching phase (15–45 min), the rate of pH change is relatively high, and the system successfully captures this change. Measurements taken at 35 °C show a pH that is slightly lower than that at 15 °C; the curves at different temperatures are distinguishable, and the results reflect the differences in response caused by temperature variations.

pH detection can be used to track the release of alkaline substances during the leaching process and can be applied to assess the environmental suitability of materials.

#### 5.2.2. Calcium Leaching

**Figure 23:**
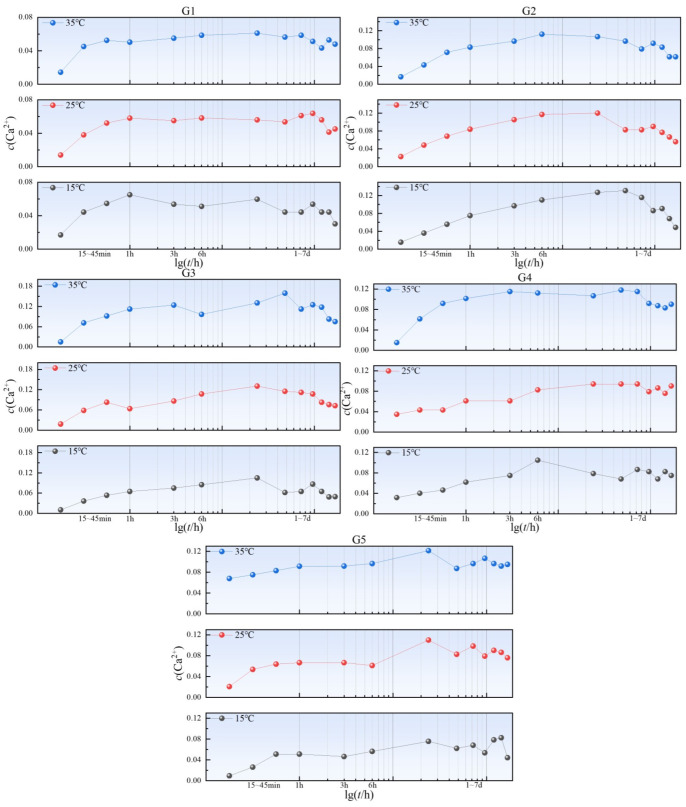
Changes in Ca^2+^ leaching concentration and soaking time.

Figure 23 shows that the Ca^2+^ concentration in all experimental groups generally exhibited a pattern of rapid initial increase, fluctuation and adjustment in the middle phase, and a localized decrease or stabilization in the later phase. In the pure cement group (G1), Ca^2+^ concentrations were primarily distributed between 0.04 and 0.06 mol/L, while in the phosphogypsum-blended groups, peak concentrations reached 0.10–0.16 mol/L—a difference of approximately 3–4 times. Subsequently, concentrations declined to varying degrees during the middle and late stages. When compared with pH changes, this phenomenon is associated with the precipitation of CaCO_3_ and Ca(OH)_2,_ resulting from liquid-phase carbonation.

The system maintained a continuous response within the concentration range of 0.04–0.16 mol/L, recording the rapid accumulation process during the first hour of leaching. During this stage, the rate of concentration change was high; continuous multi-ion detection clearly revealed the timing of peak occurrence and post-peak trends, and allowed for combined analysis with hydroxide and fluoride ions. At 35 °C, the Ca^2+^ concentration was generally higher than that at 15 °C, and the time to peak occurred earlier; the kinetic differences at different temperatures were reflected in the curves. During the detection period, the signals in each channel were stable with no significant baseline drift, indicating that the system can maintain a stable response even under conditions of higher ionic strength. The dynamic variation data of Ca^2+^ concentration also validated the system’s suitability for detection within a higher concentration range.

#### 5.2.3. Fluoride Leaching

**Figure 24:**
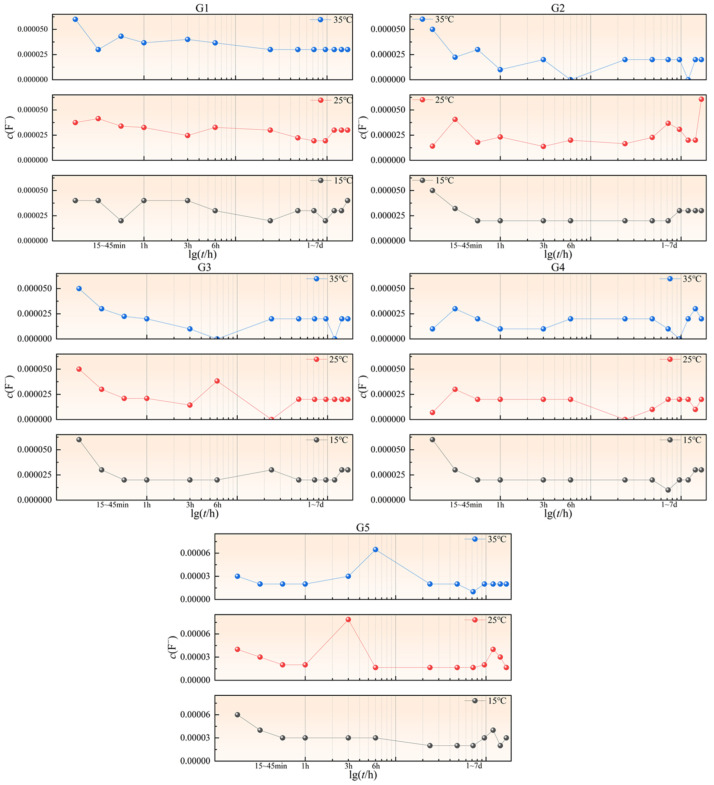
Changes in F^−^ leaching concentration and soaking time.

Figure 24 shows that the F^−^ concentration in all experimental groups generally remained at the 10^−5^ mol/L level, exhibiting a trend of rapid decline in the early stage, low-level fluctuations in the middle stage, and a slight local rebound in the late stage. During the initial leaching phase (0.25–1 h), the F^−^ concentration in some groups rapidly decreased from approximately 4.0–6.0 × 10^−5^ mol/L to about 2.0 × 10^−5^ mol/L. This was attributed to the large influx of Ca^2+^ into the liquid phase during the early stage, where F^−^ combined with Ca^2+^ to form CaF_2_, resulting in a decrease in free F^−^ concentration; subsequently, concentrations in all groups fluctuated at low levels, and by the end of the leaching test (96–168 h), slight increases appeared in some curves.

The results in Section 4 indicate that elevated Ca^2+^ reduces the activity of free F^−^ by promoting CaF_2_ precipitation; in this experiment, the trends of Ca^2+^ and F^−^ were consistent, demonstrating that the system can reflect changes in F^−^ activity under conditions of coexisting ions. During the stabilization phase, the difference in F^−^ concentration among groups was approximately 1–2 × 10^−5^ mol/L, and the system was able to effectively distinguish these differences. No persistent drift in low-concentration signals was observed during the detection period, and the curves maintained a discernible trend overall.

The F^−^ detection data can be used to evaluate the solidification effectiveness of fluoride impurities in phosphogypsum within cement-based systems and the associated environmental release risks.

#### 5.2.4. Chloride Leaching

**Figure 25:**
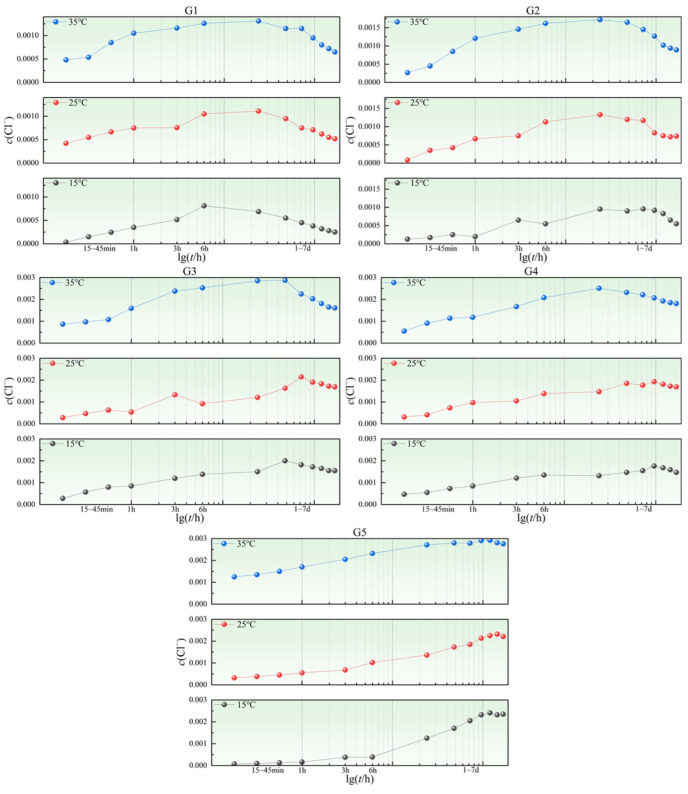
Changes in Cl^−^ leaching concentration and soaking time.

Figure 25 shows that the Cl^−^ concentrations in all experimental groups exhibited a non-monotonic trend, characterized by an initial accumulation, a peak in the middle phase, and a decline in the late phase. The peak concentration in Group G1 was approximately 0.0007–0.0013 mol/L, while that in Group G5 increased to 0.0023–0.0029 mol/L; the concentration declined after the peak, which is related to the adsorption of Cl^−^ by C–S–H gel and the consumption of Cl^−^ in the liquid phase due to Friedel’s salt formation during the continuous hydration of cement. There were significant differences in peak concentrations and time to peak among the groups with different admixture levels. Br^−^ and NO_3_^−^ were not detected in this experiment; their concentrations in this system are extremely low, and their impact on Cl^−^ detection is negligible.

Multiple measurements in the early stages fully documented the entire process of accumulation, peak formation, and post-peak decline, making the transition points at each stage clearly distinguishable. At 35 °C, peak concentrations were generally higher than those at 15 °C, and peak times occurred earlier; the effect of temperature on the Cl^−^ migration rate is clearly reflected in the curves. The curves for all groups within each cycle were continuous, with no obvious abnormal jumps.

The complete record of the Cl^−^ release process can be used to determine the peak of chloride ion release and its evolution, thereby providing a basis for durability evaluation.

The system’s time-aligned acquisition of F^−^, Cl^−^, Ca^2+^, and pH allows the interrelationships among these parameters to be directly characterized on a single timeline. The trend of rapid Ca^2+^ increase accompanied by a simultaneous decrease in F^−^ during the early leaching phase aligns with the Ca^2+^–F^−^ precipitation equilibrium mechanism and is consistent with the conclusions of the interference test in Section 4; pH measurement provides an alkalinity background reference for interpreting the activity of each ion. Compared to the independent detection of individual ions, the simultaneous collection of multiple parameters allows for the direct acquisition of inter-ionic correlation information without adding additional operational steps, demonstrating the system’s advantages in complex systems.

### 5.3. Comparison with Existing Literature and Mechanistic Implications

The leaching results obtained in this study are consistent with the main chemical trends reported for cementitious pore solutions and phosphogypsum-based cementitious materials, while providing additional time-aligned multi-ion information. Previous studies have shown that the pore solution of cement-based materials is highly alkaline and that its Ca^2+^, sulfate, hydroxide, and alkali concentrations are controlled by dissolution, hydration, and precipitation reactions [6,7,8]. In the present leaching system, the pH increased rapidly during the initial stage and then stabilized at 11.8–12.3 after 7 days. This range is lower than that commonly reported for fresh Portland cement pore solutions, which often exceed pH 13, because the present experiment measured the external leachate of crushed hardened pastes at a solid-to-liquid ratio of 1:10 rather than the original internal pore solution. In addition, phosphogypsum-derived acidic impurities may consume part of the hydroxide ions during early leaching. Therefore, the pH evolution reflects alkalinity release and buffering in the leaching solution rather than the pore solution composition alone.

The Ca^2+^ evolution also agrees with the known dissolution–precipitation behavior of cementitious systems. Calcium leaching from cement-based materials is closely related to portlandite dissolution, C–S–H decalcification, and subsequent precipitation reactions [15,16,17]. Recent studies using Ca^2+^ ion-selective electrodes have also shown that Ca^2+^ activity can be used to monitor calcium silicate complexation, C–S–H nucleation, and calcium binding under alkaline conditions [49,50]. In this study, Ca^2+^ increased rapidly during the initial leaching stage, especially in phosphogypsum-blended groups, indicating that gypsum-bearing phases supplied additional soluble calcium. The subsequent stabilization or partial decrease of Ca^2+^ can be attributed to secondary precipitation and binding reactions, including CaCO_3_ formation, Ca(OH)_2_ equilibrium, and interaction with silicate or sulfate-bearing hydration products. This interpretation is consistent with the literature showing that Ca^2+^ is not a conservative leached ion, but a reactive species controlled by hydration products and solution chemistry.

The F− results are particularly relevant to phosphogypsum utilization. Phosphogypsum is a typical acidic bulk solid waste, and fluoride-bearing impurities are one of the factors restricting its safe use in building materials, backfilling, and soil-related applications [48,51,52]. Recent work on modified phosphogypsum backfill materials has specifically emphasized that F^−^ contained in phosphogypsum can create leaching risk and that alkaline or calcium-bearing modifiers can enhance fluoride stabilization. In the present study, F^−^ remained at the 10−5 M level and decreased rapidly during the early stage when Ca^2+^ increased. This trend supports the interpretation that free fluoride activity was controlled by Ca–F precipitation or stabilization reactions. The result is also consistent with the interference test in Section 4.1.3, where elevated Ca^2+^ significantly changed the fluoride electrode response by reducing free F^−^ activity. Thus, the system successfully captured the coupled Ca^2+^–F^−^ behavior that would be difficult to explain from a single fluoride measurement alone.

The non-monotonic Cl^−^ release observed in this study is also consistent with reported chloride binding and adsorption mechanisms in cementitious systems. Existing studies have shown that C–S–H and layered double hydroxide-type phases can adsorb or bind chloride ions in simulated concrete pore solutions, thereby reducing free chloride concentration and changing the chloride-to-hydroxide ratio [53,54]. In the present leaching experiment, Cl^−^ was first accumulated and then decreased after reaching a peak. This pattern can be explained by the combined effects of early dissolution, subsequent adsorption by C–S–H, and chemical binding in AFm-type hydration products such as Friedel’s salt. The higher peak concentrations and earlier peak times at elevated temperature further indicate that temperature accelerates dissolution and ion migration during the early stage, while later binding and precipitation reactions reduce the free Cl^−^ concentration.

Compared with traditional offline methods such as pore-solution extraction, ICP-OES, titration, or ion chromatography, the present system does not aim to replace high-precision laboratory analysis [22,23,24,55]. Instead, its main advantage is the ability to record Cl^−^, Ca^2+^, F^−^, and pH in time-aligned acquisition frames under the same leaching conditions. This allows rapid Ca^2+^ release, simultaneous reduction of free F^−^, pH buffering, and non-monotonic Cl^−^ release to be interpreted on a single timeline. Therefore, the obtained results complement existing literature by providing synchronized in situ evidence for coupled ion migration and stabilization in phosphogypsum–cement solidified materials.

## 6. Conclusions

Based on an IoT architecture, a simultaneous in situ multi-ion detection system designed for cement-based liquid environments has been developed. This system integrates combination ion-selective electrodes for Cl^−^, Ca^2+^, and F^−^, along with a pH combination electrode. Leveraging an ESP32 microcontroller, dual ADS1115 analog-to-digital converters, and a voltage amplification module, it enables the continuous, synchronous acquisition of weak potentials across multiple parameters. The following conclusions can be drawn:

(1) The system features low hardware costs and high integration, enabling the time-aligned acquisition of multiple parameters. The minimum resolvable concentration reaches 10^−5^ M for Cl^−^ and F^−^, 10^−4^ M for Ca^2+^, and a high-precision linear response for pH is maintained within the range of 2–12; the relative standard deviation (RSD) for continuous testing at 0.01 M is consistently below 0.12%, demonstrating good short-term repeatability under the tested laboratory calibration conditions.
(2) The effects of temperature fluctuations on the measurement of different ions vary. The direction of the potential drift of the Cl^−^ and F^−^ combination electrodes exhibits a concentration-dependent reversal: in the high-concentration range of 0.1 M, the drift becomes negative as temperature increases, while in the low-concentration range of 0.01–0.0001 M, it shifts to a positive drift; for the Ca^2+^ combination electrode, due to the exothermic complexation mechanism of the PVC-based carrier membrane, the reading decreases monotonically as temperature rises.
(3) The effects of interfering ions on the measurement of different ions vary. At high concentrations, Br^−^ and NO_3_^−^ cause significant competitive interference with the Cl^−^ combination electrode, while Ca^2+^ reduces the activity of free F^−^ by forming CaF_2_ precipitates; the latter can be used for in situ tracking of the phase transformation and solidification behavior of fluorides in cement-based systems.
(4) The system is suitable for detecting ion leaching behavior in cement-based materials. When applied to dynamic leaching experiments on phosphogypsum–cement composite solidification bodies, the system successfully captured the multi-ion competitive synergy process involving rapid Ca^2+^ leaching, simultaneous F^−^ precipitation consumption, and non-monotonic Cl^−^ release, thereby supporting laboratory assessment of ion-leaching behavior, durability-related chemical processes, and environmental risk in solid-waste-based cementitious materials.

The proposed system provides a low-cost and time-aligned in situ sensing approach for laboratory studies of ion leaching, hydration-related solution evolution, and durability-related chemical processes in cement-based materials. In the present study, the monitoring applicability was evaluated according to short-term repeatability, practical concentration resolvability, temperature-response characteristics, ion-interference behavior, and signal stability during phosphogypsum–cement leaching tests. Specifically, the system showed RSD values below 0.12% in ten consecutive measurements, resolvable concentration limits of 10^−5^ M for Cl^−^ and F^−^ and 10^−4^ M for Ca^2+^ based on the 3σ potential uncertainty criterion, and no obvious baseline drift during the monitored leaching period. These results support its use for laboratory in situ and time-aligned multi-ion monitoring. However, unattended long-term infrastructure monitoring has not yet been fully validated in this study. Future work should further evaluate long-term electrode drift, membrane aging, embedded-service durability, reference-electrode stability, recalibration strategies, and field-scale deployment before the system is applied to long-term engineering monitoring.

## Figures and Tables

**Table 1:** Grouping of Phosphogypsum-Cement-Based Sample Formulations.

Group	Cement (g)	Phosphogypsum (g)	Phosphogypsum Content (%)
G1	80	0	0
G2	72	8	10
G3	64	16	20
G4	56	24	30
G5	48	32	40

## Data Availability

The original contributions presented in this study are included in the article. Further inquiries can be directed to the corresponding author.
